# Supplementary material for: An empirical estimation of aggregate import demand under foreign exchange constraints: Evidence from Ethiopia
Source: PLoS One. 2024 Jun 6;19(6):e0303587. doi: 10.1371/journal.pone.0303587 (PMC11156386; doi:10.1371/journal.pone.0303587)
Supplement: Attachment — Submitted filename: Reviewer comments.docx [file pone.0303587.s002.docx]

The research topic is quite interesting. I like the idea of using the problem of maximizing the consumption function over time to determine import demand. However, the author should consider the following points.

1. Literature review section: The author should provide assumptions for each model or theory.

2. Methodology section: the author should provide assumptions and clearly identify endogenous and exogenous variables.

3. Check the accuracy of formulas 3, 4, 5, 7, and 8.

4. “ln(𝑌𝑡 − 𝑃𝑡𝑀𝑡) in the right hand side of equation defined as GDP minus exports.” Should it be “GDP minus imports”?

5. The consumption maximization problem does not explain the inclusion of MSt and VOLt in the model. However, the model can be easily explained by the consumption theory provided in several macroeconomics textbooks, which states that import demand depends on income, exchange rates, and trade promotion/restriction policies.

6. The author needs to consider the endogeneity issue in the model.

7. Equation 14: lnD should be D.

8. The methodology section should mention the time length and data sources.

9. The author should provide data descriptions, correlation analysis, and tests on the model's multicollinearity and endogeneity issues.
